# Transcriptomic profiling of three-dimensional cholangiocyte spheroids long term exposed to repetitive *Clonorchis sinensis* excretory-secretory products

**DOI:** 10.1186/s13071-021-04717-2

**Published:** 2021-04-20

**Authors:** Jung-Woong Kim, Junyeong Yi, Jinhong Park, Ji Hoon Jeong, Jinho Kim, Jihee Won, Seok Chung, Tong-Soo Kim, Jhang Ho Pak

**Affiliations:** 1 Department of Life Science, College of Natural Sciences, Chung-Ang University, Seoul, 06974 Republic of Korea; 2 Department of Convergence Medicine, University of Ulsan College of Medicine and Asan Institute for Life Sciences, Asan Medical Center, Seoul, 05505 Republic of Korea; 3 School of Mechanical Engineering, Korea University, Seoul, 02841 Republic of Korea; 4 Department of Tropical Medicine and Parasitology, Inha University School of Medicine, Incheon, 22212 Republic of Korea

**Keywords:** *Clonorchis sinensis* infection, Excretory-secretory products, Three-dimensional, Cholangiocyte spheroids, Transcriptomic profiling, Microarray, RNA-Seq

## Abstract

**Background:**

Biliary tract infection with the carcinogenic human liver fluke, *Clonorchis sinensis*, provokes chronic inflammation, epithelial hyperplasia, periductal fibrosis, and even cholangiocarcinoma. Complications are proportional to the intensity and duration of the infection. In addition to mechanical irritation of the biliary epithelia from worms, their excretory-secretory products (ESPs) cause chemical irritation, which leads to inflammation, proliferation, and free radical generation.

**Methods:**

A three-dimensional *in vitro* cholangiocyte spheroid culture model was established, followed by ESP treatment. This allowed us to examine the intrinsic pathological mechanisms of clonorchiasis via the imitation of prolonged and repetitive *in vivo* infection.

**Results:**

Microarray and RNA-Seq analysis revealed that ESP-treated cholangiocyte H69 spheroids displayed global changes in gene expression compared to untreated spheroids. In ESP-treated H69 spheroids, 185 and 63 probes were found to be significantly upregulated and downregulated, respectively, corresponding to 209 genes (*p* < 0.01, fold change > 2). RNA-Seq was performed for the validation of the microarray results, and the gene expression patterns in both transcriptome platforms were well matched for 209 significant genes. Gene ontology analysis demonstrated that differentially expressed genes were mainly classified into immune system processes, the extracellular region, and the extracellular matrix. Among the upregulated genes, four genes (*XAF1*, *TRIM22*, *CXCL10*, and *BST2*) were selected for confirmation using quantitative RT-PCR, resulting in 100% similar expression patterns in microarray and RNA-Seq.

**Conclusions:**

These findings broaden our understanding of the pathological pathways of liver fluke-associated hepatobiliary disorders and suggest a novel therapeutic strategy for this infectious cancer.

**Graphic abstract:**

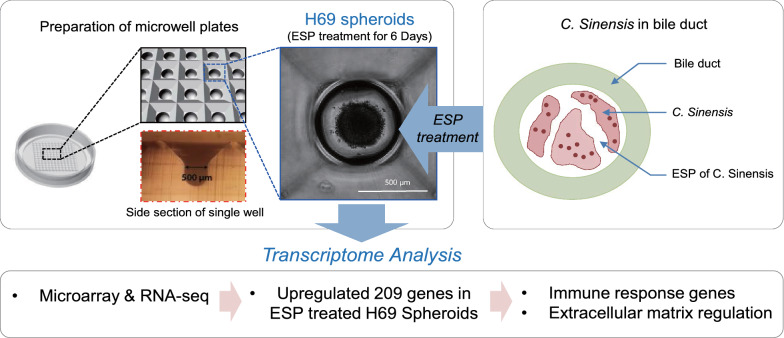

**Supplementary Information:**

The online version contains supplementary material available at 10.1186/s13071-021-04717-2.

## Background

Cholangiocarcinoma (CCA) arises from transdifferentiation and subsequent neoplastic conversion of normal cholangiocytes into malignant cells in the epithelial lining of the biliary tract. Malignant transformation of cholangiocytes is characterized as uncontrolled growth and high capacity of tissue invasiveness/metastasis [1]. Although poorly defined in detail, the molecular mechanism of cholangiocarcinogenesis is proposed as a multistep process: chronic inflammation of the bile ducts, cholangiocyte damage associated with reactive cell proliferation, consequent increases in genetic mutations and epigenetic changes, and dysregulation of apoptosis and neoangiogenesis [1]. The established risk factors for CCA include primary sclerosing cholangitis, hepatolithiasis, and choledochal cystic disease. Another critical factor is infection with liver flukes (*Opisthorchis viverrini* or *Clonorchis sinensis*), which accounts for the majority of cases of CCA in Southeast Asian countries [2, 3]. These liver flukes have been classified as definite biological carcinogens by the International Agency for Research on Cancer [4].

Proposed carcinogenic mechanisms associated with chronic liver fluke infection include mechanical injury to bile duct epithelia caused by the activities of worms, immunopathological changes due to infection-related inflammation, including secondary bacterial infection, and the pathological effects of their excretory-secretory products (ESPs), which consist of a complex mixture of proteins and other metabolites [5]. These combined physical and chemical irritations provoke inflammation, adenomatous hyperplasia, goblet cell metaplasia, periductal fibrosis, and granuloma formation, all of which contribute to malignant transformation and create vulnerability to a tumorigenic environment [6, 7].

During infection, liver flukes continuously release ESPs from the tegument and excretory openings into the bile ducts and surrounding liver tissues of the host. Cells exposed to *C. sinensis* ESPs have been used to examine the pathological processes and dysregulation that occur in infected animal models. For example, ESPs promoted proliferation via upregulation of cell cycle proteins in HEK293 cells [8] and stimulated inflammation in human CCA cells (HuCCT1) with enzymetically generated free radicals in an NF-κB-dependent manner [9]. Differential transcriptional and proteomic profiling of ESP-treated HuCCT1 cells showed that regulated genes and proteins were involved in apoptosis, carcinogenesis, metabolism, redox homeostasis, and signal transduction, demonstrating the multiple roles of ESPs in the pathological processes of host cells [10, 11]. Moreover, differential profiling of cancer-related microRNA (miRNA) expression revealed that miRNAs involved in cell proliferation and inhibition of tumor suppression were dysregulated in both CCA cells and normal cholangiocytes exposed to ESPs, indicating common ESP-responsive pathological signal cascades in both cancerous and non-cancerous bile duct epithelial cells [12].

Spheroids are self-assembled spherical clusters of cell colonies and can help span the experimental gap between monolayer cell lines and 3-dimensional organoids. The self-aggregated three-dimensional structure facilitates cell-cell and cell-matrix interactions and prevents dedifferentiation. Spheroid-based culture systems have been used for numerous applications, including tissue engineering, stem cell research, disease modeling, and as drug screening platforms [13]. Compared to traditional monolayer cell culture, multicellular spheroids better resemble native tissues in the context of structural and functional characteristics, representing conditions similar to that in an *in vivo* microenvironment [13, 14]. Multicellular tumor spheroids generated from a broad range of cancer cell lines have been reported to be highly organized tissue-like structures with homogeneous size distribution and reproducibility [15, 16]. Primary hepatocyte spheroids from rats and humans have been developed to restore liver tissue formation with metabolic functions over extended periods of long-term culture [17, 18], suggesting that the three-dimensional spheroid system is suitable for chronic hepatic abnormality studies. Moreover, it has been reported that long-term cultured spheroids generated from normal and diseased human cholangiocytes morphologically and biochemically resemble bona fide bile ducts, useful for studying various types of chronic cholangiopathic progress [19]. It is, therefore, of interest to apply these cholangiocyte spheroids to the development of *in vitro* clonorchiasis, which assimilates natural *C. sinensis* infection.

To better understand the pathological mechanisms underlying the progression and etiology of chronic clonorchiasis-associated hepatobiliary disorders, we mimicked aspects of prolonged *C. sinensis* infection by repeatedly and continuously treating human cholangiocyte spheroids with *C.* *sinensis* ESPs and assessed the time-dependent gene regulation. Utilizing microarrays containing 47,323 probes for human complementary DNAs (cDNAs) of known function and validating them with RNA-Seq, we analyzed the differential expression patterns of the transcriptomes in response to ESPs.

## Methods

### Materials

Cell culture medium components were purchased from Life Technologies (Grand Island, NY, USA) unless otherwise indicated. Polyclonal antibodies against cytokeratin 19 (Ck-19) and Ki-67 were purchased from Abcam (Cambridge, UK). All other chemicals (biotechnology grade) were purchased from Sigma-Aldrich (St. Louis, MO, USA).

### Human cholangiocyte (H69) culture

The H69 cells between the 25th and 30th passage were maintained in Dulbecco’s Modified Eagle Medium (DMEM): DMEM/F12 (1:1) containing 10% FBS, an antibiotic mixture, 1.8 × 10^−4^ M of adenine, 5 μg/ml of insulin, 5.5 × 10^−6^ M of epinephrine, 2 × 10^−9^ M of triiodothyronine, 5 μg/mL of transferrin, 1.64 × 10^−6^ M of epidermal growth factor (EGF), and 1.1 × 10^−6^ M of hydrocortisone. Cells were cultured at 37 °C in a humidified 5% CO_2_ incubator.

### Ethics statement

Animal care and experimental procedures were performed in compliance with the national guidelines outlined by the Korean Laboratory Animal Act (no. KCDC-122-14-2A) of the Korean Center for Disease Control and Prevention (KCDC). The KCDC-Institutional Animal Care and Use Committee (KCDC-IA-CUC) ethics committee reviewed and approved the ESP preparation protocols (approval identification number, KCDC-003-11). New Zealand albino rabbits (male, 10 weeks old) were infected with ~ 250 metacercariae twice (with an interval of 1 week) via intragastric intubation. After 12 weeks, *C. sinensis* adult worms were collected from sacrificed rabbit livers for the preparation of ESPs.

### Preparation of ESPs

The ESPs from *C. sinensis* adult worms were prepared as previously described [9]. In brief, adult worms were recovered from bile ducts and washed several times with cold phosphate-buffered saline (PBS) to remove any host contaminants. Five fresh worms were cultured in 1 ml of prewarmed PBS containing antibiotic mixture and protease inhibitor cocktail (Sigma-Aldrich) for 3 h at 37 °C in a 5% CO_2_ incubator. The culture fluid was then pooled, centrifuged, concentrated with a Centriprep YM-10 filter unit (Merck Millipore, Billerica, MA, USA), and filtered through a sterile 20-μm syringe membrane. Even though these ESPs contained small sizes of peptides with < 10 kDa (Additional file 1: Figure S1), small peptides functionally important for the host interaction might be still excluded during the filtering process. The protein concentration of the ESPs was measured using DC Protein Assays (Bio-Rad, Hercules, CA, USA). ESP aliquots were stored at − 80 °C until use.

### Cell seeding in three-dimensional SpheroFilm and ESP treatment

SpheroFilm microwells were obtained from InCyto Co. (Chonan, Korea). The SpheroFilm was placed at the bottom of 60-mm culture dish (Falcon, Corning, NY, USA). Before cell seeding, 100% ethanol was added to the plate and repeatedly pipetted to remove air bubbles from the wells. Once the ethanol was removed, the wells were washed with PBS and then incubated with complete culture medium at 37 °C in 5% CO_2_ for 24 h. The cells were detached from the culture dish with 0.05% trypsin/EDTA solution, counted, and adjusted to 1 × 10^6^ cells/ml. The cell suspension was poured into SpheroFilm microwells and plated on the culture dish, and the culture dish was centrifuged at 100×*g* for 3 min to settle the cells inside each section of the microwell. After cell seeding, the culture dish was washed to prevent remaining cells outside the microwells from adhering and growing. The spheroid in each microwell was fully formed at day 5 of the culture period. Fully formed spheroids were incubated with serum-/EGF-free culture (conditional) medium for 24 h before ESP treatment. ESPs at a concentration of 4 μg/ml were added into the medium, and the culture medium containing ESPs was changed every 2 days, as described for previous ESP-treated H69 three-dimensional culture [20]. Approximately 50 spheroids were harvested at 5 and 10 days after ESP treatment for extraction of total RNA.

### Immunocytochemical fluorescence

Approximately ten spheroids were collected from the microwells at the indicated time points for immunocytochemical analysis. Spheroids were washed twice with PBS, fixed with 4% paraformaldehyde for 10 min, placed in optimal cutting temperature compounds (Sakura Finetek, USA Inc., Torrance, CA, USA), and frozen using dry ice. The frozen spheroid sections (5 µm thick) were blocked with 3% bovine serum albumin (BSA) in 0.1% Triton X-100 + PBS (PBST) for 1 h, washed with PBST, and incubated overnight at 4 °C with PBS containing 1% BSA and the primary antibodies (1:50 dilution for CK-19, 1:500 dilution for Ki-67), followed by anti-rabbit secondary antibody conjugated with Alexa Fluor 488 (1:1000 dilution; Invitrogen, Carlsbad, CA, USA) for 1 h. After two washes with PBS, fluorescent spheroid images were taken using a confocal laser-scanning microscope (LSM 780; Carl Zeiss, Jena, Germany).

### RNA preparation

Total RNA from each time point was extracted using Trizol (Invitrogen) and purified using RNeasy columns (Qiagen, Valencia, CA, USA) according to the manufacturers’ protocol. After processing with DNase digestion and clean-up procedures, RNA samples were quantified, aliquoted, and stored at − 80 °C until use. For quality control, RNA purity and integrity were evaluated by denaturing gel electrophoresis (OD 260/280 ratio) and analyzed on an Agilent 2100 Bioanalyzer (Agilent Technologies, Palo Alto, CA, USA).

### Microarray analysis

Total RNA was amplified and purified using an Ambion Illumina RNA amplification kit (Ambion, Austin, TX, USA) to yield biotinylated cRNA, according to the manufacturer’s instructions. In brief, 550 ng of total RNA was reverse-transcribed to cDNA using a T7 oligo(dT) primer. Second-strand cDNA was synthesized, *in vitro* transcribed, and labeled with biotin-NTP. After purification, the cRNA was quantified using an ND-1000 spectrophotometer (NanoDrop, Wilmington, DE, USA). Seven hundred fifty nanograms of labeled cRNA samples was hybridized to each human HT-12 expression v.4 bead array for 16–18 h at 58 °C, according to the manufacturer’s instructions (Illumina, Inc., San Diego, CA, USA). Detection of array signals was carried out using Amersham FluoroLink streptavidin-Cy3 (GE Healthcare Bio-Sciences, Little Chalfont, UK), following the bead array manual. Arrays were scanned with an Illumina Bead Array Reader confocal scanner according to the manufacturer's instructions. The quality of hybridization and overall chip performance were monitored by visual inspection of both internal quality control checks and the raw scanned data. Raw data were extracted using the software provided by the manufacturer (Illumina GenomeStudio v2011.1, Gene Expression Module v1.9.0). Two independent experiments were performed to select commonly regulated genes showing good reproducibility and reliability with a mean average.

### RNA-Seq analysis

The construction of the RNA-sequencing library was performed using a TruSeq RNA Sample Preparation Kit v.2 (Illumina, Inc., cat. no. RS-122-2002). In brief, 100 ng of total RNA from each sample was exposed to poly-T oligo-attached magnetic beads to isolate poly-A mRNA following mRNA fragmentation. The cleaved RNA fragments were constructed onto a double-stranded cDNA. Then, the double-stranded library was purified using AMPure XP beads to remove all reaction components. The end repair, base addition, adapter ligation, and PCR amplification steps were performed according to the manufacturer’s instructions. Libraries were analyzed by an Agilent 2100 Bioanalyzer using a high-sensitivity DNA chip (Agilent Technologies). Then, the cDNA libraries were used for paired-end sequencing using an Illumina NextSeq 500 (Illumina, Inc.).

### Validation of microarray and RNA-Seq data by quantitative real-time (RT)-PCR

Equal amounts of 100 ng of total RNA were reverse-transcribed into cDNA using a reverse transcription system (Promega). Significantly regulated genes from the microarray and RNA-Seq were quantified using an Applied Biosystems 7500 Real-Time PCR System with Fast SYBR Green Master Mix (Applied Biosystems), in accordance with the manufacturer’s protocols. The GAPDH level was measured and used to normalize the relative abundance. Table 1 shows the primer pairs designed with the PrimerDesigner program, based on the cDNA sequences in the GenBank database. Data were analyzed using the Ct method.

**Table 1:** qRT-PCR primer sequences

Gene	Primer sequences (5′ to 3′)	Accession number
XAF1	F: CTCGGTGTGCAGGAACTGTA	NM_001353135.1
	R: CAGTGCTCCTCCATGGTTTC	
TRIM22	F: CTGTGCCTCCCTGTCGTATT	NM_006074.5
	R: TCATGGGGACTAGGCAGTTC	
CXCL10	F: CTGTACGCTGTACCTGCATCA	NM_001565.4
	R: CTTGATGGCCTTCGATTCTG	
BST2	F: CACTGTGATGGCCCTAATGG	NM_004335.4
	R: CTTCTCAGTCGCTCCACCTC	

### Bioinformatic analysis for the microarray and RNA-Seq

The entire analysis pipeline of RNA-Seq was coded using R software (ver. 3.6), which was controlled by systemPipeR (ver.1.18.2). Raw sequence reads were trimmed for adaptor sequence and masked for low-quality sequences using systemPipeR. Transcript quantification of RNA-Seq reads was performed with GenomicAlignments (ver.1.20.1) using reads aligned to Ensemble v95 *Homo sapiens* transcriptome annotation (GRCh.38.95) using Rsubread (ver. 1.24.6). The FPKM (Fragments Per Kilobase of transcript per Million mapped reads) values were calculated using the ‘fpkm’ function of DESeq2 (ver. 1.24.0) and were processed with the robust median ratio method. Transcript reads were normalized by the ‘voom’ function of Limma (ver. 3.40.6). To analyze a transcript as DE, EdgeR (ver. 3.26.7) calculates the results based on the normalized counts from entire sequence alignments. Significantly DE transcripts with fold changes greater than the raw FPKM value (> 2) and adjusted *P*-value (< 0.01) in all experimental comparisons were selected and used for further analysis. Gene annotation was added by the online database using Ensembl biomaRt (ver. 2.40.4), and visualization was performed using the R base code and the gplots package (ver. 3.0.1.1). For differentially expressed gene (DEG) sets, hierarchical cluster analysis was performed using complete linkage and Euclidean distance as a measure of similarity. Gene enrichment and functional annotation analysis for the significant probe list was performed using GO (http://geneontology.org) and DAVID (https://david.ncifcrf.gov/). All data analysis and the visualization of DEGs was conducted using R version 3.0.2 (www.r-project.org). For the statistical analysis, values were presented as mean ± SEM of three independent experiments. Data were analyzed by two-way ANOVA followed by Tukey’s multiple comparison test using GraphPad Prism software version 5.01 (California, USA). Differences between groups were considered to be significant at *P* < 0.05.

### Statistical analyses

Experimental values were presented as mean ± SEM of three independent experiments. Data were analyzed by Student’s *t*-test or two-way ANOVA followed by Tukey’s multiple comparison test using GraphPad Prism software version 5.01 (San Diego, CA, USA). Differences between groups were considered to be significant at *P* < 0.05.

## Results

### H69 spheroid formation and growth

**Fig. 1:**
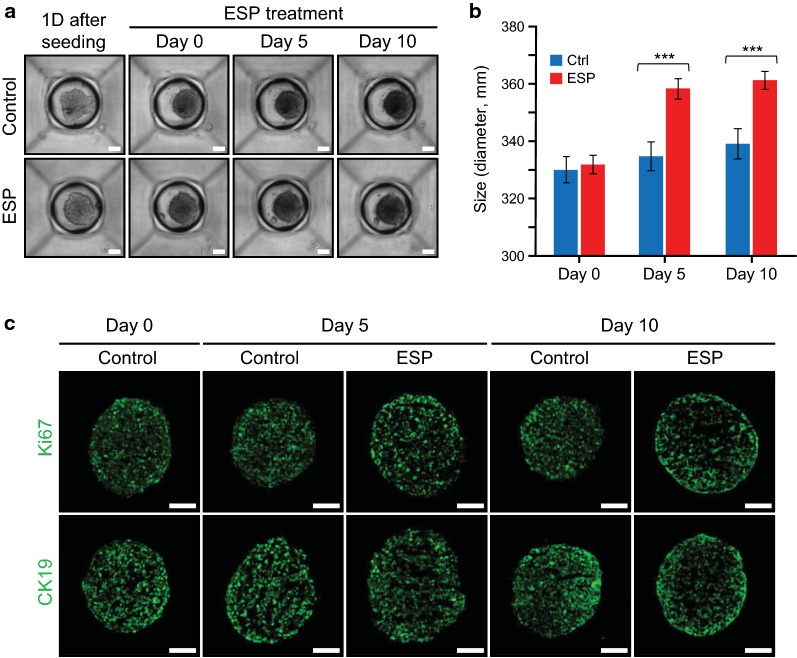
H69 cell spheroid formation in microwells and immunostaining of Ki67 and CK19. **a** H69 cells were seeded into the platform, and the cells were observed under a light microscope over a 10-day period. Scale bar = 100 μm. Original magnification: ×40. **b** Data in the graph are presented as the average size of ~ 50 spheroids at each time point. Error bars are means ± SEM. *P*-values were obtained from Student’s *t*-test. ***P < 0.001 versus untreated controls. **c** Representative immunofluorescence images of Ki67 and CK19 in the spheroids at each time point. Spheroid cryosections were incubated with Ki67 or CK19 antibodies, followed by incubation with Alexa Fluor 488-conjugated secondary antibodies. Scale bar = 100 μm. Original magnification: ×40

One day after seeding at a density of 1.5 × 10^3^, H69 cells in concave microwells (500 μm diameter) randomly migrated and adhered to each other to form cell clusters. Several small cell clusters, as well as many individual cells, gradually merged into large cell aggregates at the center of each microwell surface. This spontaneous self-aggregation of H69 cells promoted bigger and denser pre-matured spheroid formation and eventually became tightly packed cholangiocyte spheroids with an average diameter of ~ 330 μm by about day 5 (Fig. 1a). These mature spheroids were incubated with conditional culture medium for 24 h, replaced with fresh conditional medium supplemented with ESPs (4 μg/ml), and consecutively cultivated for 5 and 10 days. Cholangiocyte spheroids treated with ESPs for 5 days were significantly bigger than the untreated controls (~355 μm *vs* ~ 330 μm in diameter; increased by ~ 7.6%), and this increased size (~ 5.8%) remained almost constant up to day 10 of the experimental period (Fig. 1b). There were no significant differences in cell viability between the treated and untreated spheroids on day 0 to day 10 of culture (data not shown). Immunofluorescent staining of Ki-67 and CK-19, markers for proliferating cells and human cholangiocytes, respectively, was evenly distributed across the cell aggregates until day 10 (Fig. 1c), indicating that proliferation activity and bile epithelial characteristics were well preserved during the experimental period. Over 10 days of culture, the proliferation zone and quiescent viable cell zone of spheroids became narrow and the necrotic core was expanded (data not shown), which might mask actual ESP-mediated transcriptional regulation. Thus, subsequent analyses were performed using up to 10-day-old spheroids.

### Transcriptomic analysis of ESP-treated H69 spheroids by microarray and RNA-Seq

**Fig. 2:**
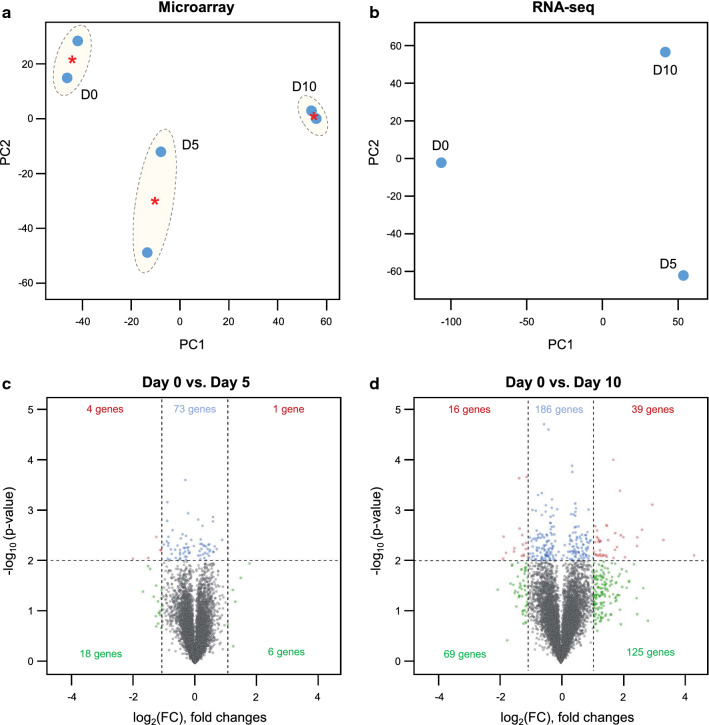
Transcriptomic dynamics by ESP treatment in H69 spheroids. Common gene expression patterns through two independent experiments and one experiment were identified by the microarray and RNA-Seq platforms **a** and **b**, respectively. Principal component analysis (PCA) of microarray (**a**) and RNA-Seq (**b**) analysis. The values indicate the amount of variation attributed to each principal component. Small blue circles indicate individual samples, red asterisks indicate the average between experimental replicates, and larger yellow ovals represent each experimental group. **c**, **d** Volcano plot of microarray of ESP-exposed H69 spheroids for 5 days (**c**) and 10 days (**d**) illustrating genes from the filtered data set that are significant DEGs between untreated- and ESP-treated H69 spheroids. Blues are significantly regulated but at less than twofold change, greens have greater than twofold change, but this is not significant, while reds are significant by more than twofold

In oncogenomic studies, PCA (principal component analysis) is generally used to analyze the complex multi-dimensional gene expression datasets. The PCA results for the microarray and RNA-Seq datasets revealed the separation of H69 spheroid groups by the exposure times of ESPs (Fig. 2a, b). The first and second principal components (PC1 and PC2) accounted for the largest variance among all datasets generated from the microarray platform (Fig. 2a). A similar assessment of the RNA-Seq dataset also showed a larger degree of separation among experimental groups (Fig. 2b). The raw data for microarray and RNA-Seq are included in Additional file 2: Table S1 and Additional file 3: Table S2. A clear segregation of ESP-treated H69 spheroid samples from their corresponding controls (day 0) was observed in both the microarray and RNA-Seq datasets, reflecting the differential gene expression patterns observed with long-term treatment of ESPs.

Application of DEGs with a conservative approach to the microarray data obtained from the ESP-exposed H69 spheroids identified 24 differentially expressed (DE) probes at day 5 (Fig. 2c) and 248 DE probes at day 10 (Fig. 2d), with the microarray signal intensity values having ≥ twofold change [FC] and ≤ 0.01 adjusted *P*-value. The 248 probes (upregulated and downregulated) from the microarray in the ESP-treated H69 spheroids are listed in Additional file 4: Table S3. For comparison with the untreated control group, volcano plots were constructed by integrating both the *P*-value and FC of each probe of the microarray data (adjusted *P*-value ≤ 0.01 and absolute log_2_ (FC) ≥ 1) to show the general scattering of the probes and to filter the DE transcripts for the ESP-treated groups for 5 days and 10 days (Fig. 2c, d). Among 248 DE probes, 63 transcripts (25.4% of DE probes) were downregulated in the H69 spheroids treated with ESPs for 10 days, while 185 probes were significantly upregulated (*P*-value ≤ 0.01 and absolute log_2_ (FC) ≥ 1) (Fig. 2d). These data suggest that transition in the transcriptome pattern is highly regulated, and specifically occurred after ESP treatment of H69 spheroids, constituting a critical step in the early stress response and physiological changes without cell death.

### Gene expression correlations between the microarray and RNA-Seq platforms

**Fig. 3:**
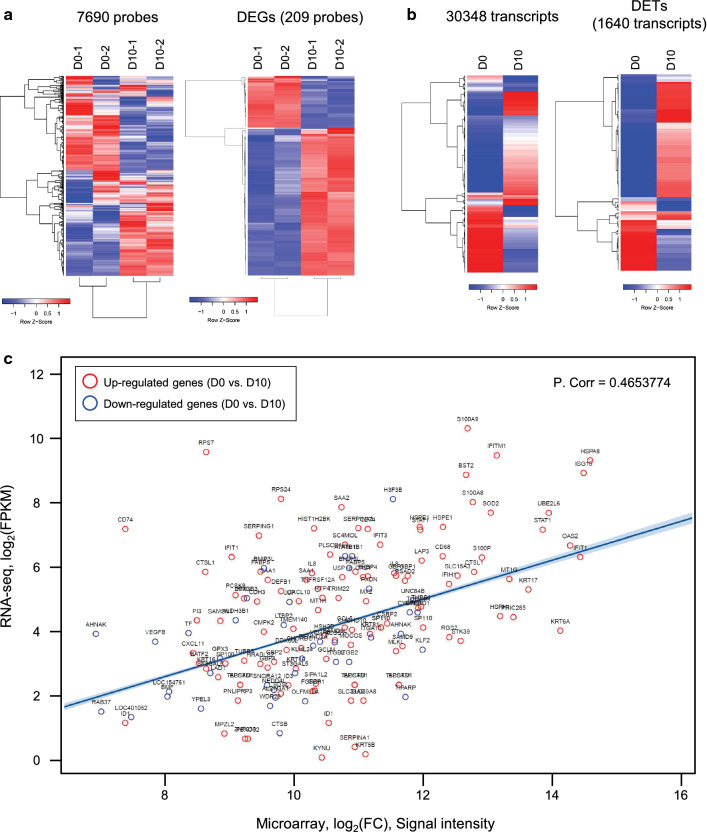
Differential gene expression patterns and correlations in the microarray and RNA-Seq analysis. **a**, **b** Hierarchically clustered genes (rows) and experimental samples (columns) with dendrograms and clusters from microarray (**a**) and RNA-Seq (**b**) data. Red in the heatmap indicates upregulation and blue denotes downregulation of gene expressions based on *z*-score. **c** Pearson’s correlation plot for 209 DEGs determined by microarray and RNA-Seq. Upregulation and downregulation patterns were 100% correlated in both platforms. Red open circle denotes upregulated genes in microarray and RNA-Seq, and blue open circle indicates downregulated genes in both platforms

The 248 statistically filtered probes (upregulated and downregulated) from the microarray data (Additional file 1: Figures S2, S3; Additional file 4: Table S3) in the H69 spheroids treated with ESPs for 10 days were used for unsupervised hierarchical clustering analysis based on Pearson’s correlation. The averaged signal intensity and log_2_ of the normalized signal intensity values of the ESP-treated and untreated control groups showed a decisive shift in the ESP exposure transcriptome in the form of upregulated and downregulated transcripts (Fig. 3a, left panel; 7690 significantly filtered probes). Among 7690 filtered probes, 248 DE probes (209 genes) were visualized by hierarchical clustering and showed significantly regulated gene expression patterns by ESP treatment (*P*-value ≤ 0.01 and absolute log_2_ (FC) ≥ 1; Fig. 3a, right panel). Furthermore, DE transcripts (DETs) were also validated by the RNA-Seq platform (Fig. 3b). Statistically filtered transcripts (30,348) from the RNA-Seq data (Additional file 3: Table S2) in the H69 spheroids treated with ESPs for 5 and 10 days were used for unsupervised hierarchical clustering analysis based on Pearson’s correlation (Fig. 3b, left panel). Transcripts that were significantly regulated by ESP treatment (1640 transcripts) were visualized with hierarchical clustering (Fig. 3b, right panel).

To identify a general linear relationship between the microarray fluorescence intensities and the corresponding RNA-Seq values for the significantly regulated transcripts, a Pearson’s correlation coefficient was computed to evaluate the data consistency between the two platforms (Fig. 3c). A quantitative comparison of the relative raw expression profile of the 248 probes presented in both platforms is shown in Fig. 3c for 10-day ESP-exposed H69 spheroids (the 248 probes are listed in Additional file 4: Table S3). Encouragingly, the measured gene abundance derived from these two different gene expression platforms showed a Pearson’s correlation of 0.465 for ESP treatment, indicating a positive concordance between the platforms. Also, all 248 probes showed similar gene expression patterns between upregulation and downregulation in both platforms, although their FC ratios were different.

### Gene ontology analysis of differentially expressed genes generated from the microarray and RNA-Seq platforms

**Fig. 4:**
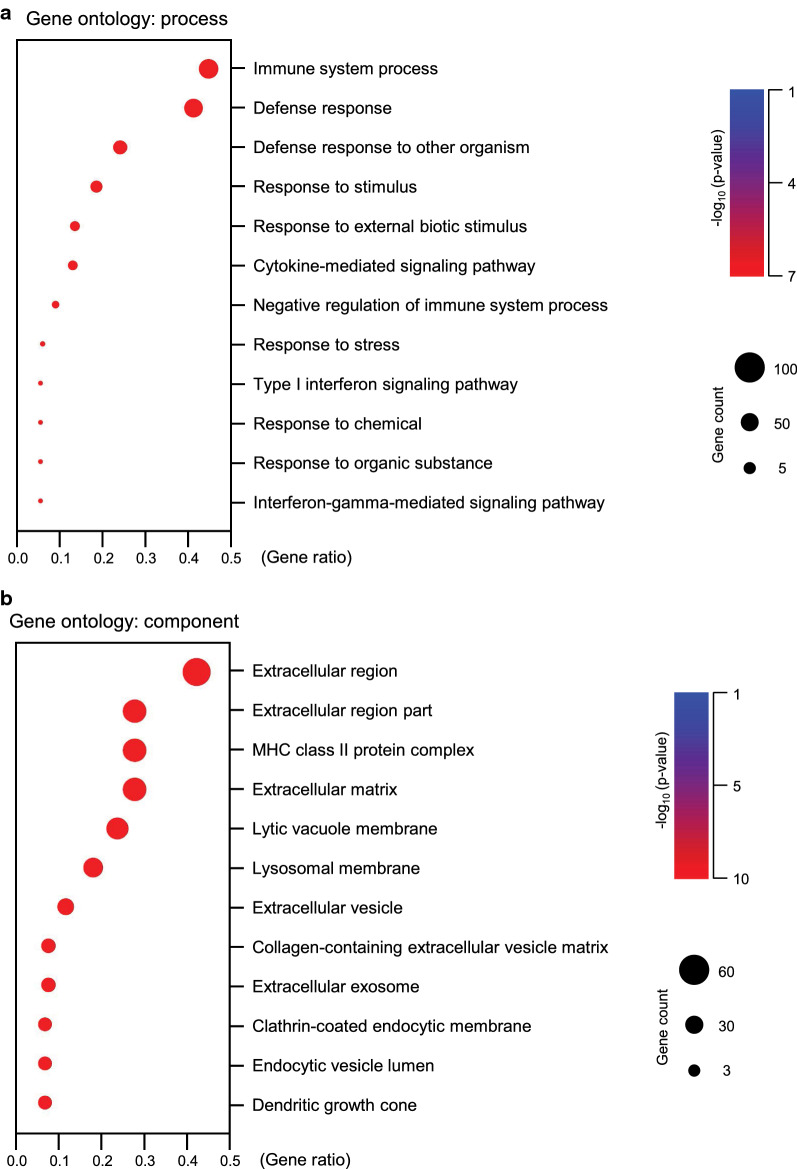
Gene ontology (GO) analysis of DEGs between untreated versus ESP-treated H69 spheroids. **a**, **b** Top enriched GO biological processes (**a**) and cellular components (**b**) of ESP-treated H69 spheroids were visualized from significant DEGs of microarray and RNA-Seq platforms. The size of the circle represents gene number, and the color represents − log_10_ of the *P*-value. Functional enrichment analysis was carried out using the ClusterProfiler package in R

The potential biological functions of common DEGs were analyzed by gene ontology analysis. The DAVID gene ontology platform (GO; https://david.ncifcrf.gov/) was employed to identify the biological associations among the 1048 DETs (overlapped transcripts were removed from 1640 DETs) induced by ESP exposure (Fig. 4). Public GO categories, including GO processes (biological process), GO molecular functions, and GO localizations (cellular component), were assessed, in which disclosure of the biological motifs associated with the expression differences was observed. For each category, results were considered statistically significant when the *P*-value < 0.001. These analyses showed the strong clustering of 1048 DETs into 312 functional categories (Fig. 4; Additional file 1: Figures S4–S9). Upregulated and downregulated genes were independently subjected to GO analysis to classify them according to their functional roles (based on their expression patterns) and not merely according to their gene names. Excluding some overlapping terms, we were able to select diverse biological process categories significantly affected by ESP treatment (Fig. 4). For upregulated genes for the cellular process, the immune system process, defense response to other organism, and response to stimulus categories were significantly clustered (*P* < 0.001). For upregulated genes for cellular components, the extracellular region, extracellular matrix (EM), and collagen-containing extracellular vesicle matrix categories were highly clustered (*P* < 0.001). DEGs specific to ESP treatment of H69 cells were significantly enriched in multiple GO biological processes and components mainly covering stress responses and EM organization.

### Validation of the microarray results with qRT‑PCR and RNA-Seq

**Fig. 5:**
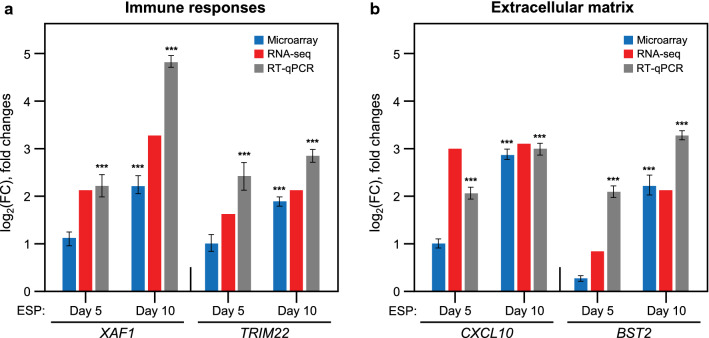
Experimental verification of gene expressions in ESP-treated H69 spheroids. **a**, **b** The gene expression profiles of immune response genes (*XAF1* and *TRIM22*) and extracellular matrix related genes (*CXCL10* and *BST2*) were verified using microarray, RNA-Seq, and qRT-PCR. The graphs show the overall fold change (FC) dynamic range (log_2_ transformed) for the indicated genes relative to those of untreated controls. For qRT-PCR, the value of each gene expression normalized by GAPDH gene represents means ± SEM for three independent experiments. ***P < 0.01 compared to untreated control of each time point

The accuracy of the gene expression estimations from the microarray analysis was validated by real-time qRT-PCR and RNA-Seq. The analysis was performed on H69 spheroids after treatment with ESPs for 5 and 10 days. A total of four upregulated genes were validated, including two immune response genes (*XAF1*: XIAP-associated factor 1 and *TRIM22*: tripartite motif containing 22) and two EM genes (*CXCL10*: C-X-C motif chemokine ligand 10 and *BST2*: bone marrow stromal cell antigen 2) in the groups treated with ESPs for 5 and 10 days in comparison to the untreated controls (Fig. 5). For the normalization of the qRT-PCR results, the *GAPDH* (glyceraldehyde-3-phosphate dehydrogenase) gene was used, based on stable mRNA expression from microarray and RNA-Seq data in ESP-treated H69 spheroids. The relative levels of gene expression in accordance with ESP exposure time were estimated as FCs, indicating significant differences for all four genes (*P* ≤ 0.01). The calculated fold changes from the microarray and RNA-Seq data were log_2_-transformed and compared with the log_2_ FCs from the qRT-PCR analysis.

### Identification of CCA-associated genes using human CCA RNA-Seq data

**Fig. 6:**
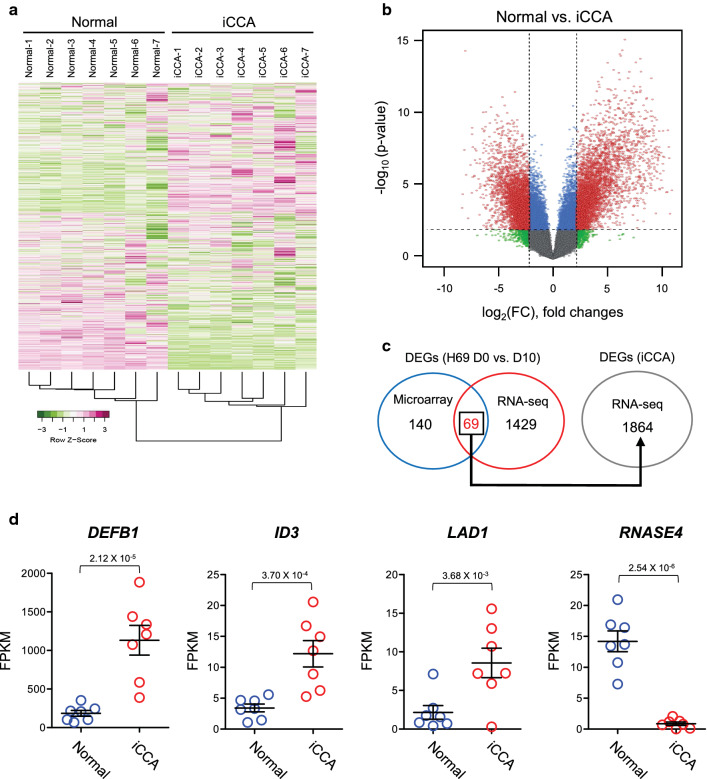
Significantly regulated gene expressions of human intrahepatic cholangiocarcinoma (iCCA) identified from microarray and RNA-Seq of ESP-treated H69 spheroids. **a** Hierarchically clustered genes (rows) and experimental samples (columns) with dendrograms and clusters for iCCA RNA-Seq data (GSE 33327). RNA-Seq raw data were reanalyzed at the transcript level. Magenta in the heatmap indicates upregulation, while green denotes downregulation of gene expressions based on *z*-score. **b** Volcano plot illustrating genes from the filtered data set that are significant DEGs between normal and iCCA. **c** Venn diagram of DEGs from ESP-treated H69 spheroids using microarray and RNA-Seq. Gene expression of 69 overlapping genes between microarray and RNA-Seq was verified in 1864 significantly regulated genes from iCCA RNA-Seq data. **d** *DEFB1*, *ID3*, and *LAD1* genes were identified as being significantly upregulated in iCCA, and *RNASE4* was identified as a downregulated gene in iCCA compared to normal tissue-matched control. Error bars are means ± SEM. *P* values were obtained from Student’s *t*-test

We further examined whether the DEGs from ESP-exposed spheroids correlated with those of human CCA samples. For this purpose, we utilized the public RNA-Seq data of intrahepatic CCA (iCCA) tissues and matched normal tissues from patients (*n* = 7) as controls provided by GEO (GSE33327) and reanalyzed these data as described in the Materials and Methods section. To filter the data, a threshold of ≥ 2 FPKM in all groups was selected as expressing transcripts, with a total of 9624 transcripts passing this criterion. Hierarchical clustering analysis of the selected 9624 transcripts was performed to identify the dynamic gene expression correlation among samples. The samples were clustered based on iCCA or normal control tissues (Fig. 6a). The volcano plot highlighted and included 1864 significant DETs with study-wide significance between normal controls and iCCA (Fig. 6b). Next, we examined the expression patterns of the common 69 DEGs from the microarray and RNA-Seq results across the patient RNA-Seq samples (Fig. 6c, gene names are listed in Additional file 5: Table S4). Among 69 common DEGs, the expressions of 48 genes were validated in the same patterns of patient data. For the stringently filtered set of 69 genes, the expressions of *DEFB1* (defensin β1), *ID3* (inhibitor of DNA binding 3), *LAD1* (ladinin 1), and *RNASE4* (ribonuclease A family member 4) were differentially regulated in iCCA tissues, compared with normal tissues (Fig. 6d). These findings indicated a high correlation between the transcriptomic dynamics of ESP-treated H69 spheroids and CCA tissues.

## Discussion

The major pathological consequences of liver fluke infection are persistent irritation and chronic inflammation of the bile duct epithelium via direct contact with worms and their ESPs. These combined actions confer the initiation, propagation, and development of more severe hepatobiliary diseases, including cholangitis, cholecystitis, cholelithiasis, and even CCA [21]. The complications associated with this infection depend on the duration of infection, worm burden, location of the worms, and the susceptibility of the host [6, 7]. In the present study, we profiled the differentially regulated gene expressions in three-dimensional cholangiocyte spheroids continuously exposed to *C*. *sinensis* ESPs and analyzed the transcriptional regulation of the genes involved in liver fluke-associated hepatobiliary disorders by mimicking the initiation and progression of prolonged infection *in vivo*. The findings indicated that ESPs exhibit pathogenic effects and extracellular matrix changes on human cholangiocyte H69 cells, subsequently leading to immune responses.

Three-dimensional multicellular spheroids from various mammalian cells display higher similarity to physiological tissues in genotypic and phenotypic aspects than monolayer cells, recapitulating better *in vivo* biological or pathological processes [13, 14]. Three-dimensional cultures of cholangiocyte organoids/spheroids have been generated from human-induced pluripotent stem cells [19], cholangiocytes of polycystin-2 defective mice [22], and cholangiocytes of normal or primary sclerosing cholangitis patients [23]. The previous studies demonstrate that these spheroids are useful models to explore the pathological mechanisms of various hepatobiliary disorders as well as being applicable for new therapeutic targets and drug screening. In the present study, the uniform sizes and biliary epithelial properties of H69 cholangiocyte spheroids were maintained in entire *C*. *sinensis* ESP treatment periods under serum-free conditions (Fig. 1). This is particularly important for transcriptome analyses, in which morphological viability differences and binding of ESP components to serum proteins may confound the interpretation of actual ESP-dependent changes in transcriptomic expression. Serum-free conditions were also reported in the spheroid culture of cholangiocyte progenitors or primary human hepatocytes, once their formations were sufficiently compact. Furthermore, transcriptomic and proteomic analyses revealed that these spheroids resembled the *in vivo* biliary epithelia and liver, respectively [19]. To our knowledge, this is the first report to apply the spheroid culture to an *in vitro* chronic helminth infection model.

Proteomic ESP analyses from *C. sinensis* adult worms revealed that abundant proteins were detoxifying enzymes (glutathione *S*-transferase and thioredoxin peroxidase), myoglobine, and a number of cysteine proteases [24]. The ESPs from liver flukes function as potent inducers of infection-related pathogenesis in host cells, inducing proliferation, inflammation, and disruption of redox homeostasis. These pathophysiological changes make cells vulnerable and create an environment for malignant transformation [8, 25]. Protein components from *O. viverrini* ESPs, including cathepsin F-like cysteine protease [26], granulin-like growth factor [27], and thioredoxin [28], have been characterized and immunohistochemically detected in biliary epithelia surrounding flukes in infected hamster livers. In particular, recombinant granulin-like growth factor accelerated repair of wounds in the skin of mice and cultured human cholangiocytes, but also promoted a carcinogenic microenvironment [29]. Its homologue was also reported in *C. sinensis* adult worms, whose overexpression induced mesenchymal characteristics of CCA and hepatocarcinoma cells via the activation of ERK-PI3K/AKT signaling pathways [30]. An immunogenic feature of *O. viverrini* ESPs appears to be their internalization in H69 cells via endocytosis pathways, contributing to immune-mediated pathogenesis [31]. Consistent with this, we present compelling evidence that *C. sinensis* ESPs promote host immune responses in cholangiocyte spheroids (Figs. 4 and 5). It is of interest to examine whether the ESPs are internalized in H69 spheroids in the same manner as they are in a two-dimensional monolayer culture. The involvement of *C. sinensis* ESPs in tumor promotion is supported by a recent finding that ESPs facilitate the acquisition of more malignant CCA cell characteristics in three-dimensional microfluidic co-culture with normal cholangiocytes [20].

The microarray and RNA-Seq are different platforms regarding calculation of gene expression. The microarray indirectly measures the fluorescence signal intensity from the hybridization between expressed transcripts and target probes, while RNA-Seq directly measures an amount of RNA transcripts by sequencing. The advantage of RNA-Seq compared to the microarray is that it provides an unbiased and quantitatively correct insight into all expressed transcripts [32]. Previous studies have compared these two transcriptomic platforms [32–35]. These studies generally indicate that RNA-Seq shows a broader dynamic range than microarrays and that the two platforms present reasonable DEGs (50–60%). In the ESP-treated H69 spheroids in this study, 185 and 63 probes were found to be significantly upregulated and downregulated, respectively, corresponding to 209 genes (*p* < 0.01, FC > 2). RNA-Seq was performed to validate the microarray results, and the gene expression patterns in both transcriptome platforms were well matched for 209 significant genes (Fig. 3). Our study evaluated the potential utility of RNA-Seq for mechanistic oncological investigations with more depth by using ESPs on three-dimensional H69 spheroids and in a context relevant to exploratory oncological studies in the pharmaceutical industry.

The GO analysis demonstrated that the DEGs were mainly classified into immune system processes, the extracellular region, and the extracellular matrix. Among the upregulated genes, four genes (*XAF1*, *TRIM22*, *CXCL10* and *BST2*) were selected for confirmation using quantitative RT-PCR, resulting in 100% similar expression patterns in microarray and RNA-Seq (Fig. 5). Among the immune response genes, we found *XAF1*, a tumor suppressor gene that inhibits the antiapoptotic effect of XIAP [36]. Tu et al. showed that *XAF1* overexpression could arrest the cell cycle in the G2/M phase, upregulate cell cycle‐associated protein p21, and downregulate the expressions of cyclin B1 and cyclin-dependent kinase 2 [37]. Interestingly, other studies have found that overexpression of *XAF1* can inhibit the tumor cell growth of lung cancer, liver cancer, and colon cancer through the induction of apoptosis and an increase in the sensitivity to radiotherapy and chemotherapy [38]. Tripartite motif containing (TRIM) family proteins have been shown to regulate antiviral innate immune responses. The TRIM22 protein has recently been revealed to inhibit hepatitis B virus (HBV) infection in humans. There have been some studies concerning TRIM22 in various human cancers. One study reported TRIM22 downregulation in breast cancer cell lines and tissues compared with non-malignant mammary epithelial cell lines and normal breast tissues [39]. Another study compared the transcriptional levels of TRIM family genes in non-small cell lung cancer and normal bronchial epithelial cell lines [40]. TRIM22 was one of ten TRIM genes upregulated in a cancer cell line. Thus, the function and status of TRIM22 in human cancer cells are somewhat vague and might be tissue-specific [41]. Epithelial to mesenchymal transition (EMT) is a vital process in the conversion of early stage tumors into invasive malignancies, and it has been shown that EMT is associated with lung cancer invasion and metastasis [42]. TRIM22 promotes the invading ability of lung cancer cells via the downregulation of E-cadherin and upregulation of N-cadherin [43]. Exogenous CXCL10 inhibited bleomycin-induced fibrosis and reduced the migration of fibroblasts in response to alveolar lavage fluid. The CXCL10 knockout mice also showed increased pulmonary fibrosis [44]. Embedment of cancer cells in the basement membrane matrix reveals that downregulation of BST2 shows invadopodia formation, extracellular matrix degradation, and subsequent cell invasion [45]. In the context of CXCL10 and BST2 roles during liver fluke infection, they may participate in the host defense mechanism such as the immune response at the early stage of the infection. Their continuous dysregulations at the chronic stage, however, may promote cell adhesion/migration, which contributes to the progression of liver fluke-associated hepatobiliary disorders.

Here we found significant DEGs using the public iCCA RNA-Seq data (Fig. 6). The transcriptions of three genes (*DEFB1*, *ID3*, and *LAD1*) were upregulated in iCCA, and the expression of *RNASE4* was significantly downregulated in iCCA. *DEFB3* has been shown to be upregulated by HBV, and this upregulation substantially prevented intrauterine transmission of HBV to infants [46]. As *DEFB1* has been shown to be upregulated in cirrhotic liver disease [47], we found that the expression of *DEFB1* was significantly increased in ESP-treated H69 spheroids. Consistent with our findings, differential transcriptom profiling in mouse liver infected with *C. sinensis* metacercariae revealed that the expressions of genes involved in immune responses and cell-cycle regulation were modulated [48]. Recent DEGs from the bile ducts of rats infected with *C. sinensis* have also shown the dysregulation of signal transduction pathways participating in defense responses [49].

## Conclusions

Continuous mechanical and chemical irritation during chronic *C*. *sinensis* infection can cause severe hepatobiliary disorders, including CCA. Using microarray and RNA-Seq, dynamic transcriptome analyses were performed in three-dimensional cholangiocyte spheroids repeatedly exposed to *C*. *sinensis* ESPs, mimicking chronic *in vivo* infection. We found some dynamic transcriptional changes and related gene-centered functional ontology, including immune system processes, cytokine-mediated signaling pathways, and the extracellular matrix. Our findings enable an understanding of the pathological pathways that underlie the etiology of *C. sinensis*-associated CCA, and this information provides a novel target for early diagnosis, chemoprevention, and treatment of this fatal bile duct cancer.

## Supplementary Information

**Additional file 1: Figure S1.** Silver-stained gel image of ESPs after SDS-PAGE. Lane M: MW markers; lane 1: 4 μg of ESPs. **Figure S2.** Normalization plot of microarray data. **a** Before and after quantile normalization. *Y*-axis shows log_2_ of microarray signal intensity and *X*-axis shows samples that were included for this study (H69 spheroids) and HuC cells. **b** Signal density plot designated the overall expression patterns in each sample and shows matched density distribution after quantile normalization. **c** Number of significantly detected probes showing *P*-value < 0.05 in all groups. **Figure S3.** Pearson’s correlation plot showing the similarities among groups. The matched correlation plot was generated between samples. Upper right numbers designate the correlation coefficiency and left below graphs shows the gene expression patterns between two different samples. **Figure S4.** Gene ontology analysis and enriched pathway for immune responses. Gene ontology analysis shows the critical biological pathways of the immune responses. GO-related and assigned gene names are shown in the colored box. The GO plot and overall analysis was performed by using the differentially expressed genes between the day 0 and 10 ESP-treated H69 spheroid groups. **Figure S5.** Gene ontology analysis and enriched pathway for cytokine-related pathway. Gene ontology analysis shows the critical biological pathways involved in the cytokine-mediated signaling pathway. GO-related and assigned gene names are shown in the colored box. The GO plot and overall analysis was performed by using the differentially expressed genes between the day 0 and 10 ESP-treated H69 spheroid groups. **Figure S6.** Gene ontology analysis and enriched pathway for immune system process. Gene ontology analysis shows the critical biological pathways involved in immune system process. GO-related and assigned gene names are shown in the colored box. The GO plot and overall analysis was performed by using the differentially expressed genes between the day 0 and 10 ESP-treated H69 spheroid groups. **Figure S7.** Gene ontology analysis and enriched pathway for expanded immune system process. Gene ontology analysis shows the critical biological pathways involved in the immune system process. GO-related and assigned gene names are shown in the colored box. The GO plot and overall analysis was performed by using the differentially expressed genes between the day 0 and 10 ESP-treated H69 spheroid groups. **Figure S8.** Gene ontology analysis and enriched pathway for response to stimulus. Gene ontology analysis shows the critical biological pathways involved in response to stimulus. GO-related and assigned gene names are shown in the colored box. The GO plot and overall analysis was performed by using the differentially expressed genes between the day 0 and 10 ESP-treated H69 spheroid groups. **Figure S9.** Gene ontology analysis and enriched pathway for response to ESPs. Gene ontology analysis shows the critical biological pathways involved in response to ESPs. GO-related and assigned gene names are shown in the colored box. The GO plot and overall analysis was performed by using the differentially expressed genes between the day 0 and 10 ESP-treated H69 spheroid groups.

**Additional file 2: Table S1.** Microarray data for ESP-treated H69 spheroids.

**Additional file 3: Table S2.** RNA-Seq data for ESP-treated H69 spheroids.

**Additional file 4: Table S3.** Differentially expressed genes in ESP-treated H69 spheroids.

**Additional file 5: Table S4.** Common 69 DEGs from microarray and RNA-Seq results across the patient RNA-Seq samples.

## Data Availability

The data supporting the conclusion of this article are provided within the article and its additional files. The original datasets analyzed in this study were deposited in NCBI, GEO (accession number: GSE168792).

## Abbreviations

CCA: Cholangiocarcinoma
ESPs: Excretory-secretory products
Ck-19: Cytokeratin 19
EGF: Epidermal growth factor
PBS: Phosphate-buffered saline
BSA: Bovine serum albumin
qRT-PCR: Quantitative real time-polymerase chain reaction
FPKM: Fragments per kilobase of transcript per million mapped reads
DEG: Differentially expressed gene
GO: Gene oncology
PCA: Principal component analysis
FC: Fold change
DET: Differentially expressed transcript
ECM: Extracellular matrix
XAF1: XLAP-associated factor 1
TRIM22: Tripartite motif containing 22
CXCL10: C-X-C motif chemokine ligand 10
BST2: Bone marrow stromal cell antigen 2
GAPDH: Glyceraldehye-3-phosphate dehydrogenase
GEO: Gene expression omnibus
DEFB1: Defensin β1
ID3: Inhibitor of DNA binding 3
LAD1: Ladinin 1
RNASE4: Ribonuclease A family member 4
