# Supplementary material for: Transcriptomic profiling of three-dimensional cholangiocyte spheroids long term exposed to repetitive Clonorchis sinensis excretory-secretory products
Source: Parasit Vectors. 2021 Apr 20;14:213. doi: 10.1186/s13071-021-04717-2 (PMC8056535; doi:10.1186/s13071-021-04717-2)

Additional File: Figure S1

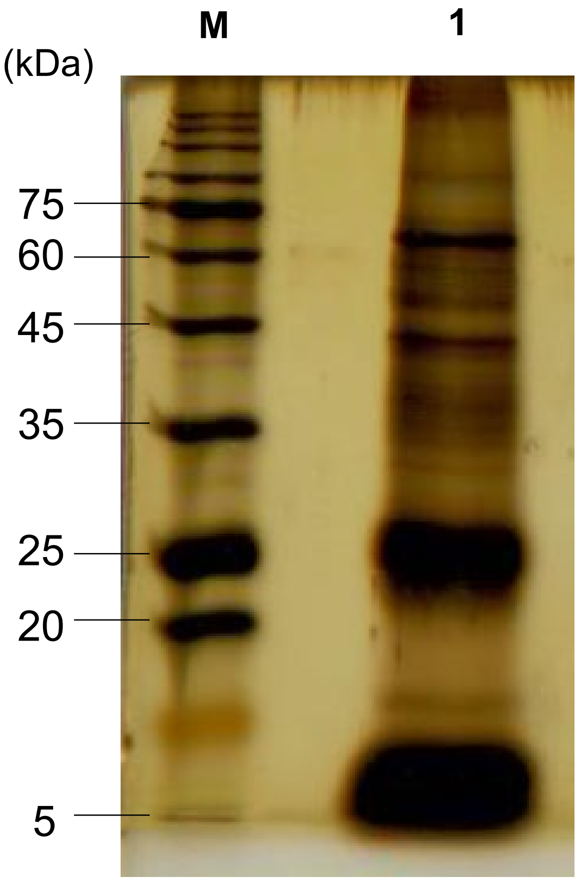

Additional File: Figure S2

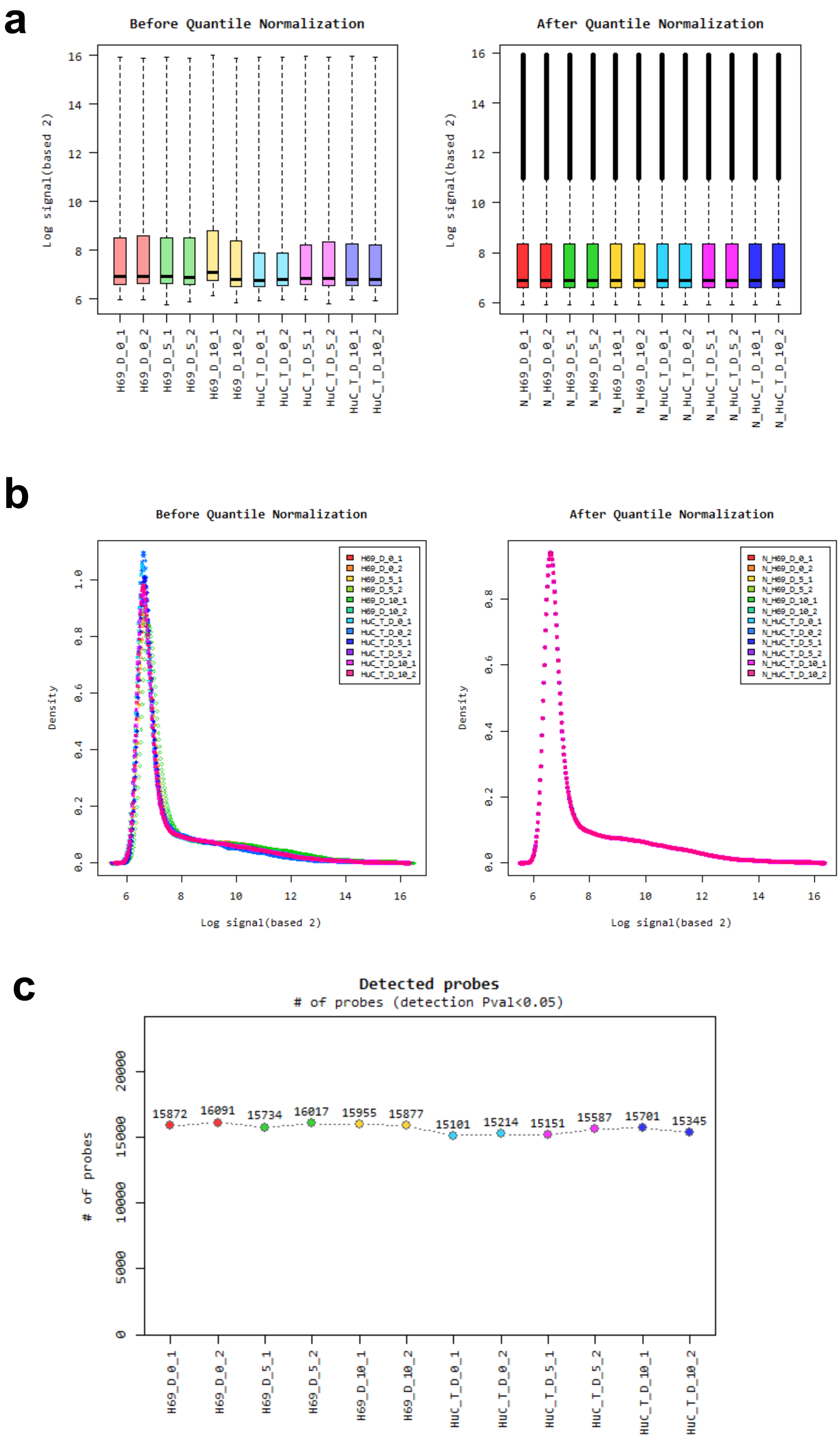

Additional File: Figure S3

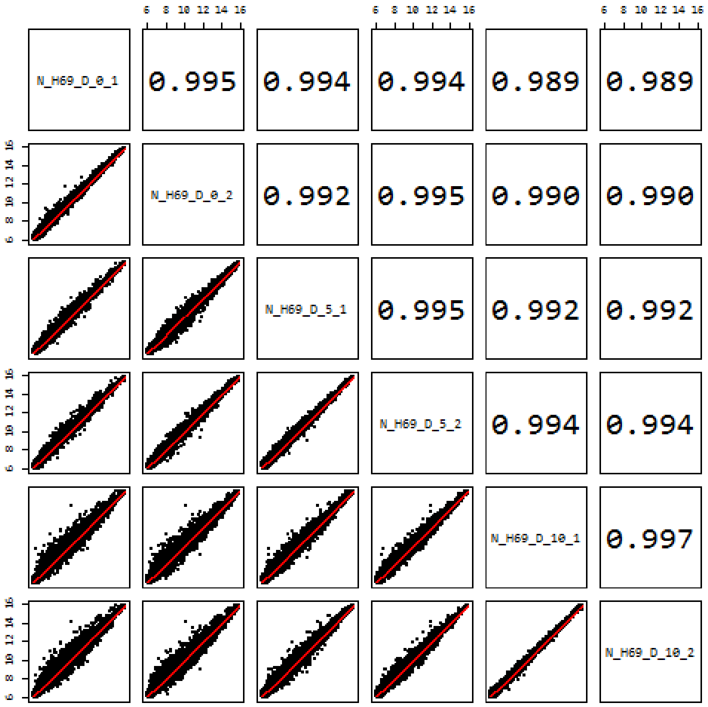

Additional File: Figure S4

Common at D10 - Process

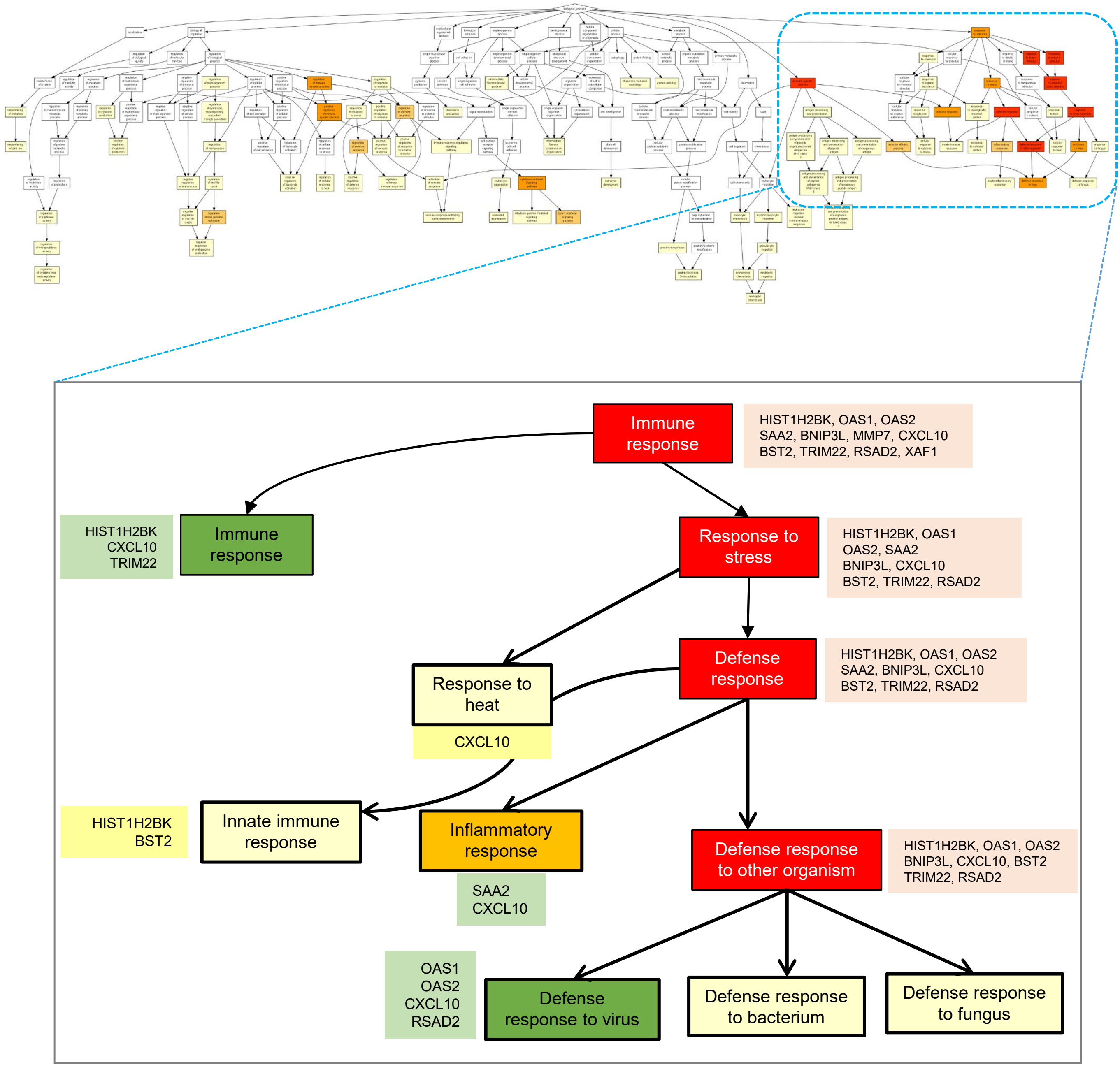

Additional File: Figure S5

H69 D0 vs D10 - Process

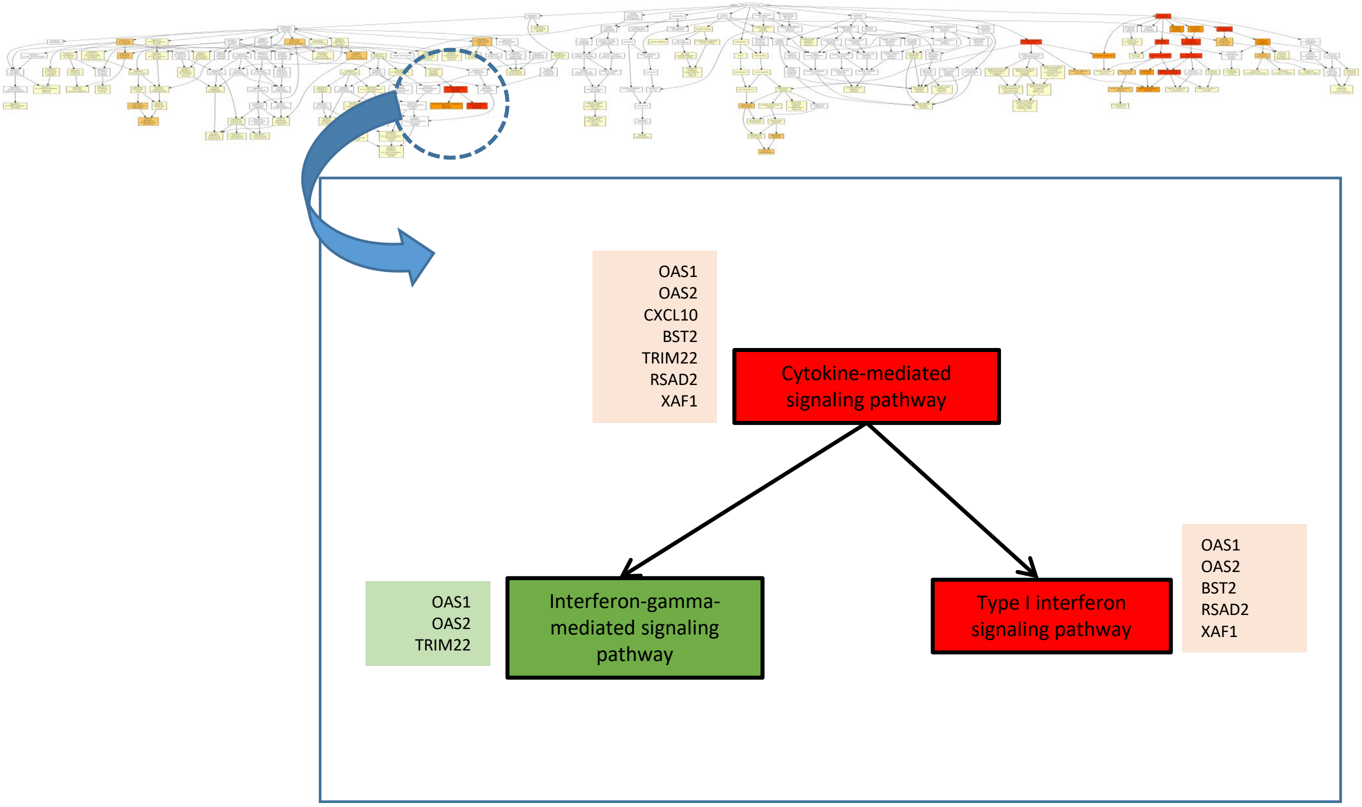

Additional File: Figure S6

H69 D0 vs D10 - Process

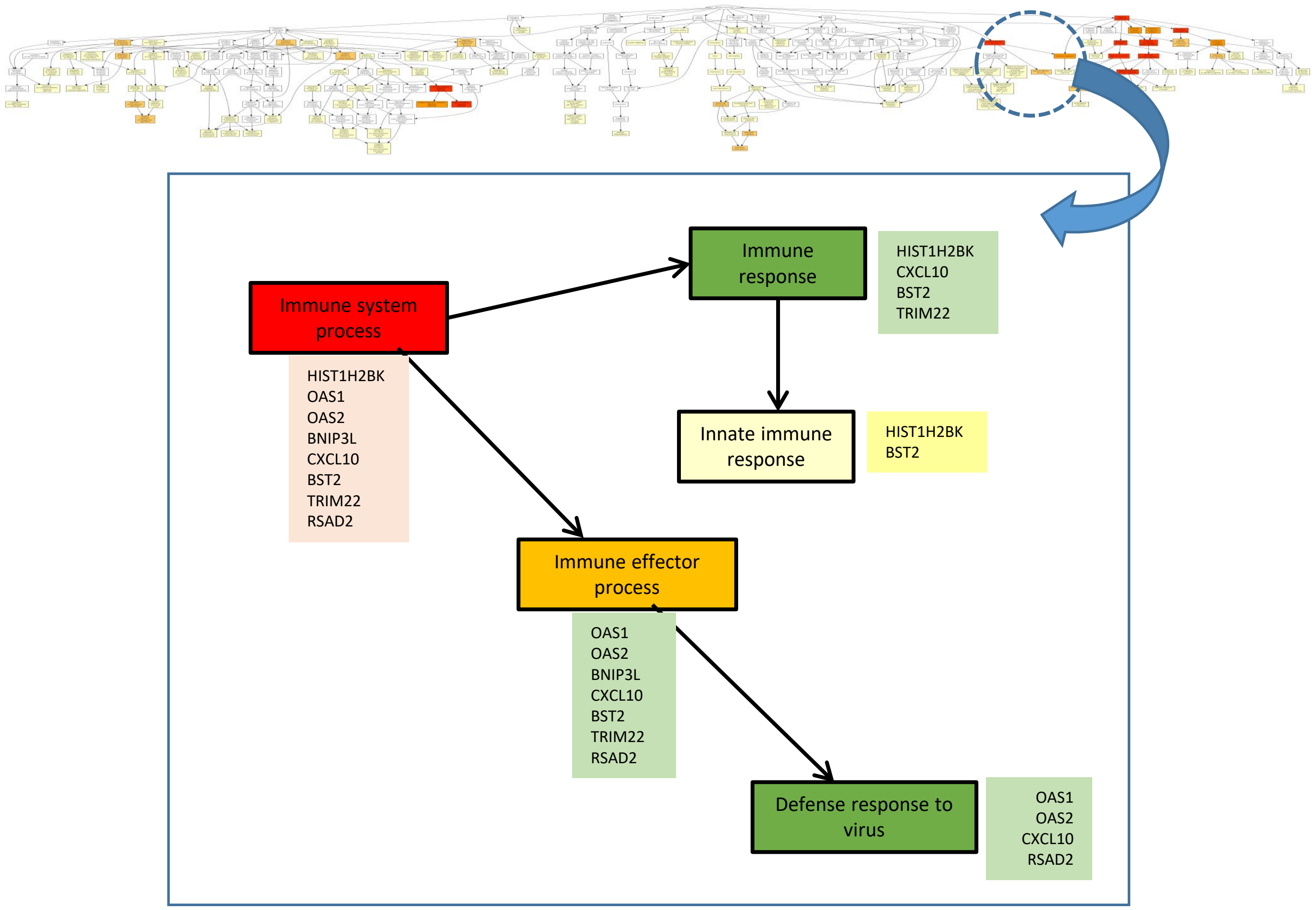

Additional File: Figure S7

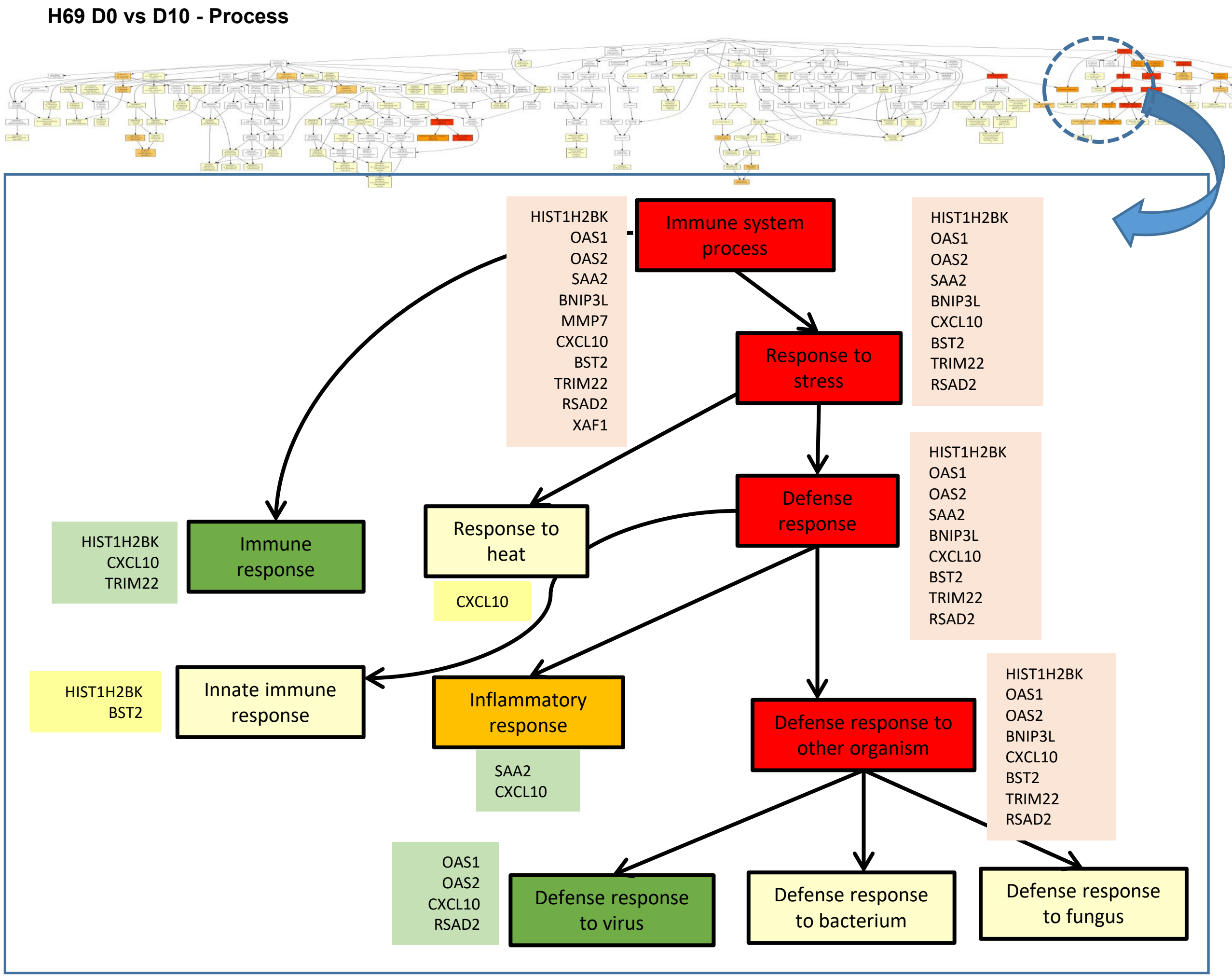

Additional File: Figure S8

H69 D0 vs D10 - Process

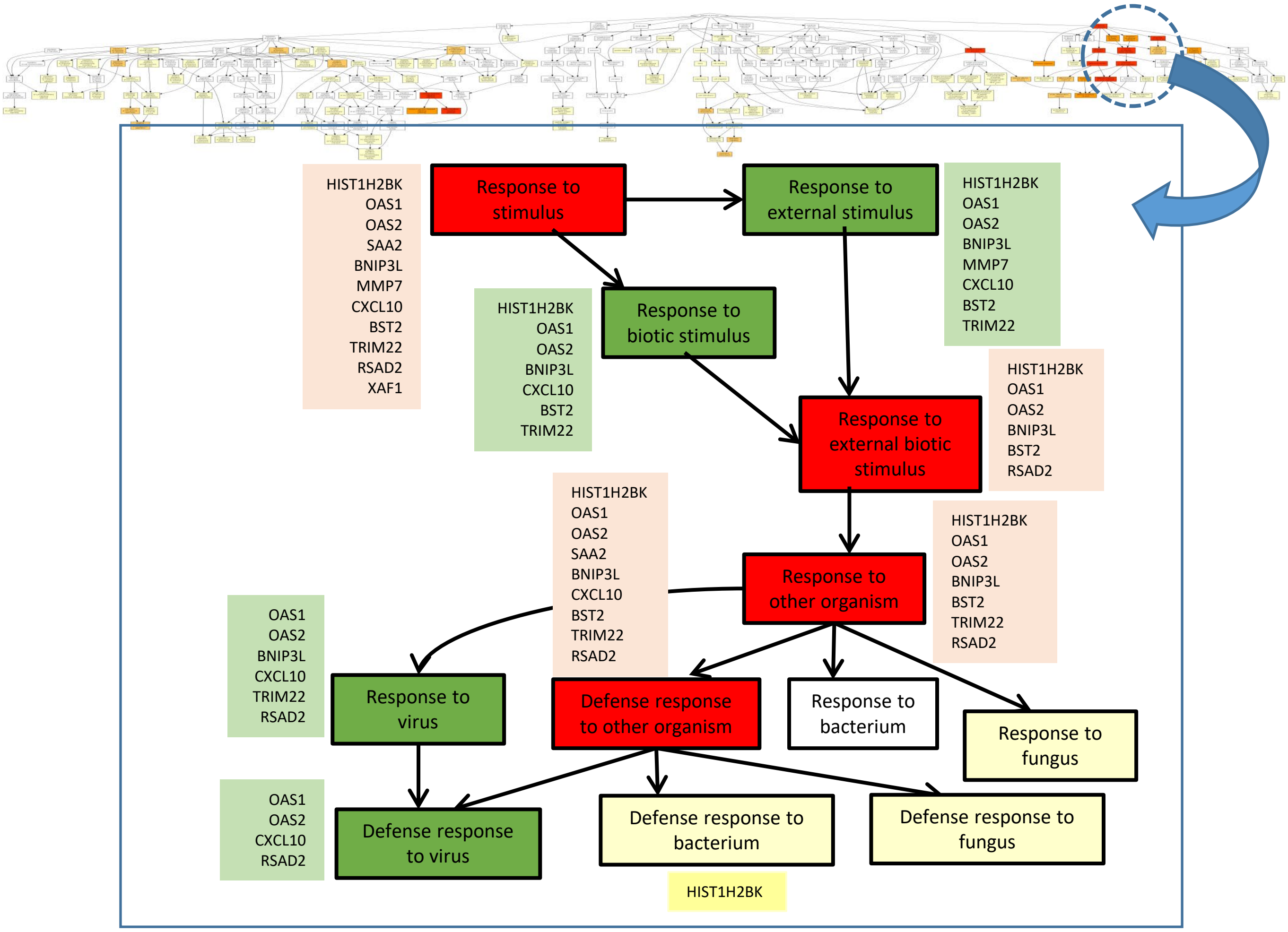

Additional File: Figure S9

H69 D0 vs D10 - Process

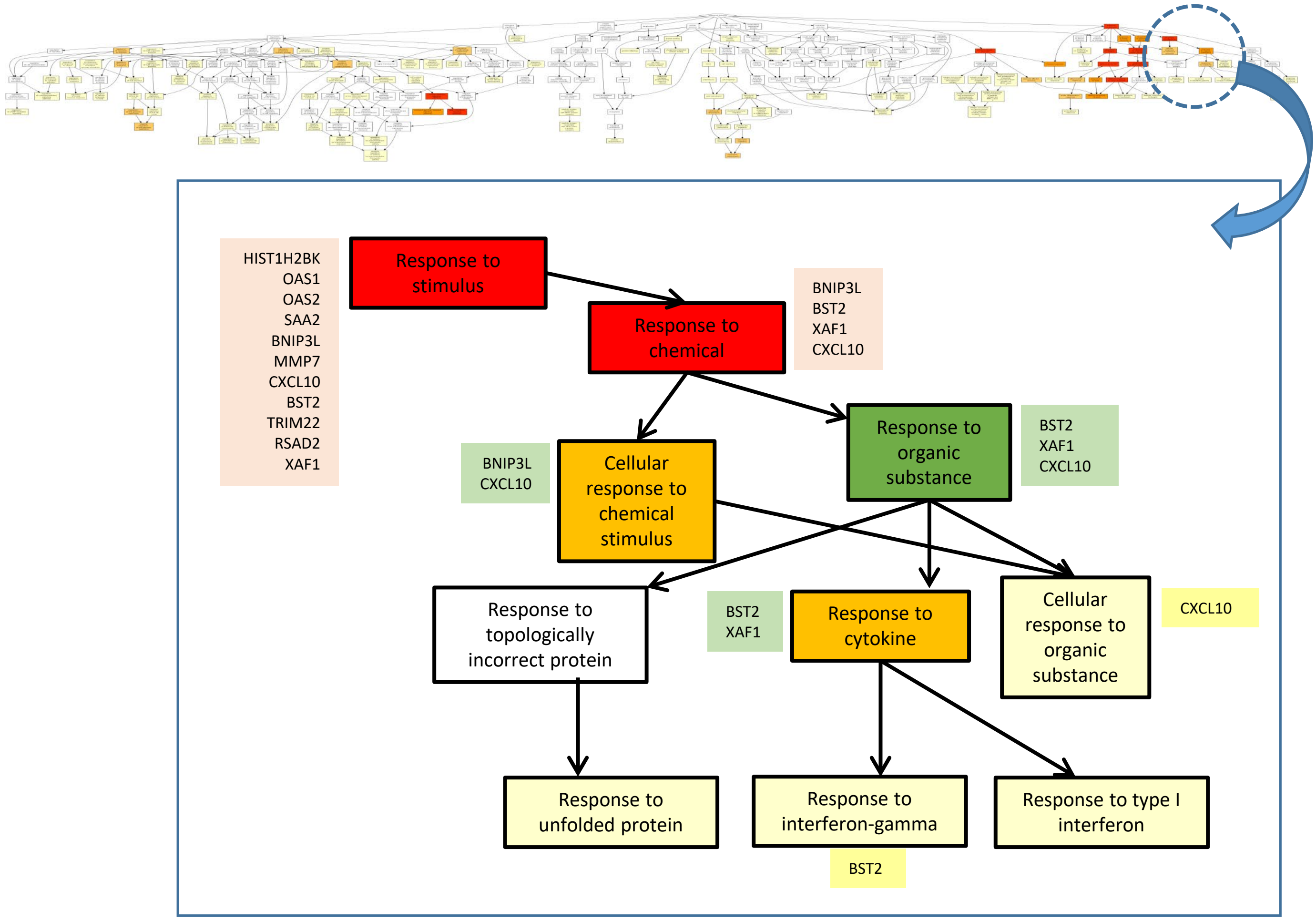

Supplement: Supplementary file 1 — Additional file 1: Figure S1. Silver-stained gel image of ESPs after SDS-PAGE. Lane M: MW markers; lane 1: 4 μg of ESPs. Figure S2. Normalization plot of microarray data. a Before and after quantile normalization. Y-axis shows log2 of microarray signal intensity and X-axis shows samples that were included for this study (H69 spheroids) and HuC cells. b Signal density plot designated the overall expression patterns in each sample and shows matched density distribution after quantile normalization. c Number of significantly detected probes showing P-value < 0.05 in all groups. Figure S3. Pearson’s correlation plot showing the similarities among groups. The matched correlation plot was generated between samples. Upper right numbers designate the correlation coefficiency and left below graphs shows the gene expression patterns between two different samples. Figure S4. Gene ontology analysis and enriched pathway for immune responses. Gene ontology analysis shows the critical biological pathways of the immune responses. GO-related and assigned gene names are shown in the colored box. The GO plot and overall analysis was performed by using the differentially expressed genes between the day 0 and 10 ESP-treated H69 spheroid groups. Figure S5. Gene ontology analysis and enriched pathway for cytokine-related pathway. Gene ontology analysis shows the critical biological pathways involved in the cytokine-mediated signaling pathway. GO-related and assigned gene names are shown in the colored box. The GO plot and overall analysis was performed by using the differentially expressed genes between the day 0 and 10 ESP-treated H69 spheroid groups. Figure S6. Gene ontology analysis and enriched pathway for immune system process. Gene ontology analysis shows the critical biological pathways involved in immune system process. GO-related and assigned gene names are shown in the colored box. The GO plot and overall analysis was performed by using the differentially expressed genes between [file 13071_2021_4717_MOESM1_ESM.pdf]
